# Impact of Spiritual Wellbeing in Advanced Cancer Patients Receiving Genomic Test Results

**DOI:** 10.1002/pon.70471

**Published:** 2026-04-23

**Authors:** Megan C. Best, Joseph Descallar, Grahame Simpson, Christine E. Napier, Nicci Bartley, Mandy L. Ballinger, David Goldstein, David M. Thomas, Katherine M. Tucker, Phyllis Butow

**Affiliations:** ^1^ University of Notre Dame Australia Institute for Ethics and Society Sydney NSW Australia; ^2^ Ingham Institute for Applied Medical Research Liverpool NSW Australia; ^3^ South West Sydney Clinical Campuses School of Clinical Medicine UNSW Sydney Liverpool NSW Australia; ^4^ Faculty of Medicine and Health University of Sydney Sydney NSW Australia; ^5^ Centre for Molecular Oncology Faculty of Medicine & Health University of NSW Sydney NSW Australia; ^6^ Psycho‐Oncology Co‐Operative Research Group (PoCoG) University of Sydney Sydney NSW Australia; ^7^ Faculty of Medicine & Health University of NSW Sydney NSW Australia; ^8^ Prince of Wales Medical School Hereditary Cancer Centre University of New South Wales Prince of Wales Hospital Randwick NSW Australia

**Keywords:** anxiety, cancer, comprehensive genomic testing, depression, FACIT‐sp‐12, fear of cancer progression, genomics, hope, spiritual wellbeing

## Abstract

**Objective:**

Little is known about the impact of treatment availability on the spiritual wellbeing of advanced cancer patients. The aim of this study was to examine the relationships between spiritual wellbeing and patient anxiety, depression, hope, and fear of cancer progression, depending on whether advanced cancer patients were able to access novel therapies.

**Methods:**

Australian adult advanced cancer patients who had exhausted all therapeutic options were recruited to undergo comprehensive genomic profiling (CGP) to determine whether a novel treatment was available. Questionnaires were administered prior to testing and within 2 weeks of receiving CGP results. Spiritual wellbeing was measured using the FACIT‐Sp‐12. Structural equation modeling was used to examine the predictive and mediating relationships among spiritual wellbeing at both time points, mediated by anxiety, depression and hope subscales at time 1 on the fear of cancer progression.

**Results:**

Both questionnaires were completed by 397 patients. A total of 238 (60%) patients had actionable CGP results. At baseline, the spiritual wellbeing constructs of meaning, peace and faith were identified as explanatory variables. At time 1, measures of spiritual wellbeing (meaning, peace and faith), and of hope, anxiety and depression, were identified as mediating variables for the outcome of fear of cancer progression. The test results were non‐significant in the model.

**Conclusions:**

The level of spiritual wellbeing in advanced cancer patients undergoing CGP was not impacted by test results but influenced a number of patient outcomes. Efforts to support patient spiritual wellbeing in such cohorts is recommended.

## 1 Background

Spirituality in the context of healthcare has been described as the way people engage with the purpose and meaning of human existence, and the way this informs their personal values [1]. It should not be confused with religion, which constitutes explicit beliefs and formal, organized practices which is just one way of expressing universal human spirituality [2].

Cancer patients report that spirituality is an important resource for coping with the physical, psychological and social challenges inherent to the disease trajectory [3, 4, 5, 6, 7]. Research has shown that spiritual wellbeing is a significant, unique contributor to quality of life for this cohort, independent of physical, social and emotional factors, and that low levels of spiritual wellbeing are associated with existential distress [5, 8, 9]. Religion and spirituality have also been found to have a potentially negative impact, for example when disease is interpreted as divine punishment [10, 11], indicating the complexity of the relationship between spiritual wellbeing and health outcomes for cancer patients. Levels of spiritual wellbeing are known to fluctuate [12], and the precipitants for reduction of spiritual wellbeing are not clear [13].

Patients with advanced cancer who have exhausted all their treatment options may experience spiritual distress as death appears imminent and may require help to address concerns related to meaning in life [13, 14]. One way of coping is to continue hoping for a cure, even in advanced disease [15]. Improved understanding of tumorigenesis has led to the introduction of comprehensive genomic profiling (CGP) to identify molecular characteristics of tumor tissue which can be targeted with inhibitor drugs which may extend therapeutic options for cancer patients who have exhausted standard treatment [16]. Because genomic results are invested with considerable hope by advanced cancer patients [17], they can also intensify spiritual concerns [18, 19, 20].

Spiritual concerns may be expressed as fear of cancer recurrence or progression [21], which is known to be a highly prevalent problem among cancer patients [22], associated with a range of negative psychosocial outcomes [22]. Interventions aimed at lowering this fear to manageable levels are increasingly sought [23]. Little is known about the impact of spiritual wellbeing, and of CGP and novel treatment availability, on fear of cancer recurrence, and whether they represent possible avenues for future intervention.

The aim of this study was to examine the relationship between spirituality and ability to access novel therapies on advanced cancer patients' fear of cancer progression, and whether these relationships are mediated by psychological wellbeing (levels of anxiety, depression and hope). We hypothesised that patients with treatment options following CGP would have higher spiritual wellbeing and lower fear of cancer progression than patients who received negative test results.

## 2 Methods

### 2.1 Participants

The Molecular Screening and Therapeutics (MoST) Program is a national cancer genomic study recruiting adult (≥ 18 years) Australian patients with pathologically confirmed advanced or metastatic solid cancers. To be enrolled in the MoST program, patients need accessible tissue which is sufficient for CGP; to either be receiving the last line of treatment or to have exhausted therapeutic options; and have an Eastern Cooperative Oncology Group (ECOG) Performance Status of 0–3 [24].

Participants are referred by their community oncologist. The MoST Program performs CGP on a patient's tumor tissue and if there is a relevant therapeutic trial available through the MoST program for actionable findings, participants are offered enrollment. The participant's oncologist receives the results and supervises treatment choices. If there is a relevant therapeutic trial available through the MoST Program for actionable findings, participants are referred by their treating oncologist.

The Psychosocial issues in Genomic Oncology (PiGeOn) Project was a longitudinal, mixed‐methods sub‐study of the MoST Program investigating the psychosocial, ethical, and behavioral implications of CGP [25]. Consent to participate in the PiGeOn Project was given while consenting to the MoST Program. Ethical approval was received from the St Vincent's Hospital Human Research Ethics committee, Sydney, Australia (Reference HREC/16/SVH/23).

The PiGeOn Project collected questionnaire data from all participants at baseline; within two weeks of receiving results (8–10 weeks after enrollment); and two months later. A qualitative study of the impact of receiving CGP results for this cohort has been previously published [26]. This paper reports on data collected at the first and second timepoints (pre and post result‐receipt).

### 2.2 Measures

The following measures were collected at baseline:

Demographic variables, including sex, age, educational level, cultural and linguistic background, socioeconomic status, country of birth, postcode, previous visit to a family cancer clinic, and parental status.

CGP result: Participants undergoing CGP receive one of three results: (1) actionable, with a clinical trial available through MoST (actionable – MoST sub‐study); (2) actionable, no clinical trial available through MoST (actionable – other treatment); or (3) no actionable variant (NAV).

The following measures were collected at baseline and T1:

Spiritual wellbeing: The Functional Assessment of Chronic Illness Therapy – Spiritual Scale‐12 (FACIT‐Sp‐12) [27, 28] measures spiritual wellbeing, with either 2 or 3 subscales: peace, meaning (combined or separated) and faith. High scores indicate greater spiritual wellbeing. Originally developed and validated on samples from the USA, the scale has had wide international use.

Fear of cancer progression: Three items from the Concerns about Recurrence Questionnaire [29], were adapted to measure fear of cancer progression (FCP), a primary outcome for the study. High scores indicate greater fear.

The following measures were collected at T1 only:

Anxiety and depression: The 14‐item Hospital Anxiety and Depression Scale [30] comprises two 7‐item sub‐scales measuring anxiety and depression. High scores indicate greater morbidity.

Hope: The 12‐item Herth Hope Index [31] measures hope and sense of meaning, with 3 subscales: temporality and future, positive readiness and expectancy, and interconnectedness. High scores indicate greater hope.

### 2.3 Statistical Analysis

Descriptive statistics were used to summarize the data using means and standard deviations for continuous variables and frequencies and percentages for categorical data. Patients who completed information at both baseline and T1 time points were included in the study.

The measurement of the spiritual wellbeing subscales of peace, meaning and faith were modeled for an Australian cohort using item response theory (IRT) examining measurement invariance and structural invariance over time using Mplus v8.5 [32]. Weighted least squares mean and variance adjusted (WLSMV) limited‐information estimation including a probit link and the THETA parameterization was used. We tested whether a 3‐factor model, consisting of peace, meaning and faith, was a more appropriate fit than a 2‐factor model consisting of peace and meaning together, and faith.

IRT, including the use of modification indices, was conducted to test the three‐factor FACIT‐Sp‐12 model: Peace (sp1, sp4R, sp6, sp7); meaning (sp2, sp3, sp5, sp8R); faith (sp9, sp10, sp11, sp12) for an Australian population.

Structural equation modeling (SEM) was used to examine the predictive and mediating relationships among spiritual wellbeing at both time points, mediated by anxiety, depression and hope subscales at time 1 on the fear of cancer recurrence. Model fit was examined by Root mean squared error of approximation (RMSEA) and Standardized root mean square residual (SRMR) with < 0.08 considered a good fit for both indices; alongside the Comparative Fit Index (CFI) and Tucker‐Lewis Index (TLI) (values > 0.90 indicating a good fit).

## 3 Results

Demographic information is listed in Table 1. A total of 1000 patients were enrolled at baseline, and 397 patients had complete information at both time points (39.7%). Older patients and patients with a cultural and linguistically diverse background were less likely to have complete information (*p* = 0.0206 and *p* = 0.0021, respectively). See Supporting Information S1 for demographic characteristics of excluded participants.

**TABLE 1:** Demographics (*n* = 397).

Characteristic	Mean (sd)	Positive test result	Negative test result	*p*‐value
Age (mean, SD)	57 (13.8)	57.6 (14)	56.5 (13.6)	0.4429

	***n* (%)**	***n* (%)**	***n* (%)**	***n* (%)**
Sex				
Male	176 (44.3)	77 (46.1)	99 (43)	0.609
Female	221 (55.7)	90 (53.9)	131 (57)	
Education level				
Primary school	4 (1)	2 (1.2)	2 (0.9)	0.9924
Year 7 or 8	17 (4.3)	7 (4.2)	10 (4.4)	
Year 9 or 10	74 (18.6)	31 (18.6)	43 (18.7)	
Year 11 or 12	59 (14.9)	24 (14.4)	35 (15.2)	
University	172 (43.3)	75 (44.9)	97 (42.2)	
Vocational training	71 (17.9)	28 (16.8)	43 (18.7)	
Cultural and linguistically diverse background (CALD)^a^				
Yes	63 (15.9)			
No	334 (84.1)			
Socioeconomic status (SEIFA)				
1 (lowest)	38 (9.6)	18 (10.8)	20 (8.7)	0.3645
2	67 (16.9)	21 (12.6)	46 (20)	
3	72 (18.1)	32 (19.2)	40 (17.4)	
4	58 (14.6)	27 (16.2)	31 (13.5)	
5 (highest)	162 (40.8)	69 (41.3)	93 (40.4)	
ARIA				
Major city	277 (69.8)	110 (65.9)	167 (72.6)	0.222
Inner regional	76 (19.1)	36 (21.6)	40 (17.4)	
Outer regional	39 (9.8)	17 (10.2)	22 (9.6)	
Remote	5 (35.7)	4 (2.4)	1 (0.4)	
Previous family cancer clinic visit				
Yes	45 (11.3)	24 (14.9)	21 (9.5)	0.111
No	337 (84.9)	137 (85.1)	200 (90.5)	
Children				
Yes	316 (79.6)	125 (74.9)	191 (83)	0.0805
No	79 (19.9)	41 (24.6)	38 (16.5)	
NA	2 (0.01)	1 (0.6)	1 (0.4)	

^a^ Culturally and linguistically diverse (CALD) describes individuals with backgrounds, languages, or religions different from the dominant culture.

The mean age of patients was 57 (SD 13.8), 55% of patients were female (*n* = 221), 43.4% had university level education (*n* = 172), 40.8% of patients in the highest socioeconomic status quintile (*n* = 162), 69.8% living in a major city area (*n* = 277), and 79.6% (*n* = 316) with at least 1 child.

Descriptive information for the baseline, mediating and outcome variables is listed in Table 2. Seventy‐one (17.9%) patients had actionable CGP results with treatment via MoST, 167 (42.1%) patients had an actionable CGP result with treatment via another pathway. Mean scores and standard deviations for the mediating and outcome variables are displayed in Table 2. While a proportion of participants fell into the distress range for anxiety and depression, the mean score was below the clinical cut‐offs. However, the averages were elevated compared to the general population. The FACIT‐sp12 scores show minimal change from baseline to time 1. Pearson correlations between the subscales indicate very weak (0 – 0.19) or weak (0.2 – 0.39) relationships between the scales at their respective time points (Supporting Information S1). The correlations for the subscales across time points show a moderate relationship (0.4 – 0.59) for Meaning (*r* = 0.56), and strong relationships (0.6–0.79) for Peace (*r* = 0.64) and Faith (*r* = 0.79).

**TABLE 2:** Explanatory, mediating and outcome variables (*n* = 397).

Variable	
CGP, *n* (%)	
No actionable variant	159 (40.1)
Treatment via MoST substudy	71 (17.9)
Treatment via other pathway	167 (42.1)
Mediating variables	
Hope (time 1), mean (SD)	38.3 (5.8)
HADS anxiety (time 1), mean (SD)	7 (4.4)
HADS depression (time 1), mean (SD)	5 (3.9)
Outcome variables	
Fear of cancer progression (baseline), mean (SD)	16.5 (7.9)
Fear of cancer progression (time 1), mean (SD)	16.7 (8.1)
FACIT subscales	
Meaning (baseline), mean (SD)	14.2 (2.3)
Peace (baseline), mean (SD)	10.5 (3.5)
Faith (baseline), mean (SD)	7.9 (5)
Meaning (time 1), mean (SD)	13.4 (3)
Peace (time 1), mean (SD)	10.7 (3.6)
Faith (time 1), mean (SD)	7.7 (5.2)

### 3.1 Factor‐Structure of the FACIT‐Sp‐12

The IRT models suggested a 3‐level factor model, containing subscales of meaning, peace and faith was a better fit than the 2‐factor model (*p* < 0.0001). However, the model fit indices of RMSEA = 0.139 and SRMR = 0.08 indicated poor model fit. Better model fit was achieved after item Sp12 was removed from the faith subscale (RMSEA = 0.068; SRMR = 0.051; CFI = 0.998; TLI = 0.997). For FACIT‐Sp subscale frequencies of response see Supporting Information S1.

Invariance testing for the spirituality subscales from baseline to time 1 revealed Sp8 in the meaning subscale to be invariant with respect to loadings and all its thresholds, as well as Sp1 in the peace subscale with all loadings and thresholds invariant.

At baseline, good reliability (> 0.8) was observed for each of the spirituality constructs subscale in patients from ‐3SD to 0 SD of the average meaning subscale (see Supporting Information S1); −2.4 SD to 1 SD of the average peace subscale (Supporting Information S1); and −1.2SD to 1.2 SD of the average faith subscale (Supporting Information S1).

## 4 SEM

**FIGURE 1:**
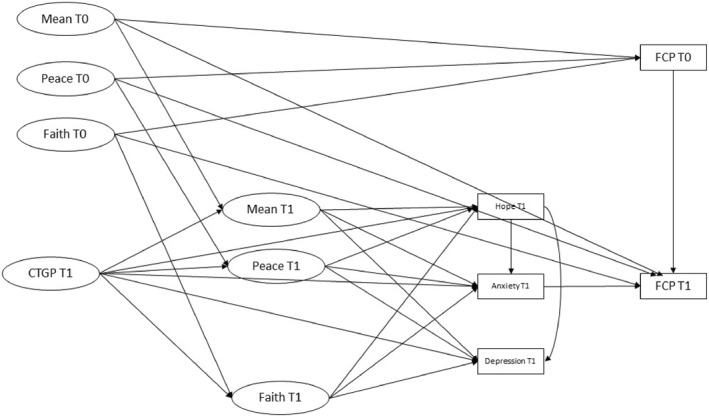
Theoretical model of spirituality in advanced cancer patients. The Functional Assessment of Chronic Illness Therapy – Spiritual Well‐Being (FACIT‐Sp) measures are as follows: Mean = Meaning subscale; Peace = Peace subscale; Faith = Faith. CTGP = Comprehensive tumor genomic profiling (actionable or non‐actionable result); Anxiety and Depression are derived from the ‘Hospital Anxiety and Depression Scale’; Hope was derived from the ‘Herth Hope Index’; FCP = Fear of cancer progression from the concerns about recurrence questionnaire.

A theoretical model was developed outlining the association between genomic pathways testing results, Spiritual well‐being (11‐item version) at baseline and time 1, the mediating variables of Hope at time 1, HADS anxiety and depression at time 1 and FCP at both time points (Figure 1).

**FIGURE 2:**
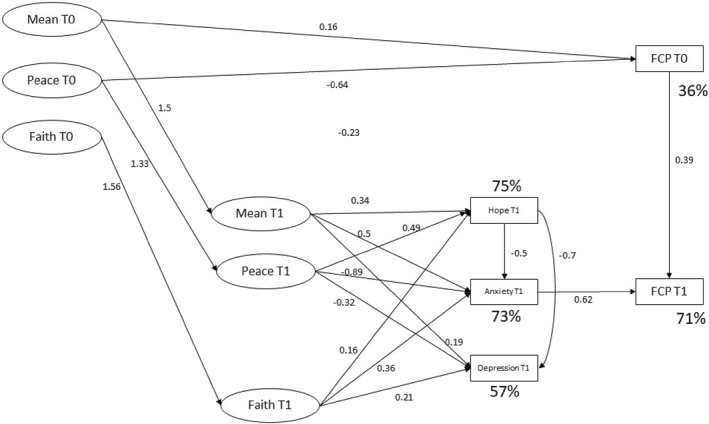
Final model of spirituality in advanced cancer patients based on structural equation model. All significant paths with their coefficients are shown. Percent (%) represents variance explained. The Functional Assessment of Chronic Illness Therapy – Spiritual Well‐Being (FACIT‐Sp) measures are as follows: Mean = Meaning subscale; Peace = Peace subscale; Faith = Faith subscale. CTGP = Comprehensive tumor genomic profiling (actionable or non‐actionable result); Anxiety and Depression are derived from the ‘Hospital Anxiety and Depression Scale’; Hope was derived from the ‘Herth Hope Index’; FCP = Fear of cancer progression from the concerns about recurrence questionnaire.

A SEM analysis was conducted to test the theoretical model against the research data. An optimal model was derived by an iterative process of trimming non‐significant path coefficients from the model until only significant (*p* < 0.05) and theoretical relevant paths remained in the model. The final model (Figure 2) fitted the data well indicated by the goodness of fit indices (RMSEA = 0.07; SRMR = 0.063; CFI = 0.98; TLI = 0.98).

At baseline, the spirituality constructs of meaning, peace and faith were identified as explanatory variables. Baseline FCP and time 1 variables of the spirituality constructs (meaning, peace and faith), hope, HADS anxiety and depression were identified as mediating variables for the outcome of FCP at time 1.

The genomic pathway testing results were non‐significant in the model. The model accounted for 36% of variance for the mediating variables of FCP at baseline. Baseline peace was associated with decreased levels of FCP at baseline (beta = −0.64). There was a positive relationship between the baseline and T1 in each of meaning (beta = 1.41), peace (beta = 1.33) and faith (beta = 1.56). Meaning at baseline had a small but positive association with FCP at baseline (beta = 0.16).

The model accounted for 71% of FCP at time 1 with a significant pathway from baseline FCP (beta = 0.39) to FCP T1. The model accounted for 73% of Anxiety at time 1 (beta = 0.62) with a strong pathway between Anxiety and FCP T1. In contrast, Depression was a co‐morbid psychological state but not related to FCP either directly, nor indirectly through Anxiety. The model also accounted for 75% of the variance in Hope, with Hope playing a buffering role with both Anxiety and Depression.

All three subscales of time 1 Spiritual wellbeing had significant pathways to Hope, Anxiety and Depression. Meaning at time 1 had positive pathways associated with elevated levels of Hope (beta = 0.34), Anxiety (beta = 0.5) and Depression (beta = 0.19). In contrast, Peace at time 1 was positively associated with increased Hope (beta = 0.49) but had inverse relationships with Anxiety (beta = −0.89) and Depression (beta = −0.25), suggestive of a buffering role. Finally, Faith demonstrated positive pathways with Hope (beta = 0.16), Anxiety (beta = 0.36), and Depression (beta = 0.21).

## 5 Discussion

In this study we used SEM to investigate whether the spiritual wellbeing of advanced cancer patients undergoing CGP with the desire to find novel therapeutic options, and results received, impacted FCP. Overall the model accounts for significant proportions of the variance in FCP T1 as well as Hope, Anxiety and Depression.

Contrary to our study hypothesis, the genomic pathway testing results were non‐significant in the model. Several reasons could account for this finding. It could be that, since patients were only eligible if standard therapeutic options had been exhausted, participants had already reconciled themselves to the possibility that no more treatment would be available. A previous study of this cohort showed that, even when positive results were available, accessing treatment was often not feasible (due to advanced disease) and therefore the result alone was not necessarily an indication of further treatment availability [26]. Adams and colleagues also found that psychological dimensions of breast cancer patients remained stable over the duration of CGP [33].

It is not unexpected to find that both FCP at baseline and broader anxiety at T1 are associated with FCP, as both are expressions of the distress that can be experienced in face of the real and ongoing threat to mortality [34]. In their study of prostate cancer patients, Parker and colleagues found that anxiety was a significant predictor of FCP [35].

In this cohort, depression was not co‐related to FCP. This may be explained by the fact that, on average, the mean HADS depression score was within the normal range (HADS 0–7), so depression was not a clinically significant feature for this sample overall. Furthermore, Hope was acting as a protective factor in relation to depression. The powerful role of hope in buffering depression in cancer patients is well documented [22, 36]. While evidence regarding the relationship between anxiety and hope remains unclear, we found that hope played a protective role in our model, and while we found no direct relationship between hope and FCP, an indirect buffering effect was mediated through this pathway.

The variables of the spirituality factors (meaning, peace and faith), hope, anxiety and depression for our sample were identified as mediating variables for the outcome of FCP at baseline and time 1. Identification of the inverse association between spiritual wellbeing and fear of cancer recurrence (FCR) has also been previously identified [37, 38, 39]. We are not aware of any other studies which have identified that the relationship is mediated by these psychological constructs. However, the pathway is not straightforward.

Peace at T0 behaved as expected, with a buffering effect on FCP at both time points, mediated through hope and anxiety at T1. Meaning was associated with increased FCP at T0, and both meaning and faith were associated with increased levels of hope, anxiety and depression at T1. As all factors of spiritual wellbeing were associated with hope, its very robust buffering effect on anxiety (0.89) means that the overall impact of spiritual wellbeing is helpful. Hope has been previously identified as having a positive relationship with spiritual wellbeing, and is boosted by supporting patients' spiritual resources [9, 40, 41, 42].

It is unclear why meaning and faith both contributed to anxiety and depression. Hundreds of studies have examined the relationship between depression and religion and spirituality [8]. While findings are mixed, the majority of studies report an inverse relationship. Raised anxiety levels have been associated with higher spirituality in some studies examining response to stressful life events [43, 44]. It is not clear whether increased spirituality caused anxiety, perhaps due to issues such as guilt, or the reverse, with spirituality recruited as a way of coping with anxiety. It is possible that the mixed emotions are a realistic appraisal of the situation in which this cohort finds themselves.

In this cohort of Australian patients with advanced cancer, we found that the three‐factor model for the FACIT‐Sp‐12 was a better fit than the original two‐factor model, as previously noted [5, 28]. We found that a better model fit for the FACIT‐Sp‐12 was achieved after item Sp12 was removed from the faith subscale. This has been found in another study of Australian patients [45]. Item 12 is a measure in the faith subscale, ‘I know that whatever happens with my illness, things will be okay.’ Item 12 is distinct from the other questions in the Faith subscale: (9) ‘I find comfort in my faith or spiritual beliefs’; (10) ‘I find strength in my faith or spiritual beliefs’; and (11) ‘My illness has strengthened my faith or spiritual beliefs’. This item does not refer to spirituality or faith directly, and contaminates the scale by capturing level of optimism, which is an aspect of mental wellbeing [46]. Item 12 also focuses on the future, rather than the present, which for advanced cancer patients may have more negative connotations than for many other cohorts. In view of our findings, we recommend that the FACIT‐Sp‐11 be used in Australian medical patients, as a clinical measure of spiritual wellbeing.

In view of the overall positive impact of spiritual wellbeing on the quality of life and coping ability of advanced cancer patients [5], cancer care would benefit from adopting the biopsychosocial spiritual model [47], and spiritual care should be routinely available in that context, particularly interventions which promote peace [48]. A recent review of existential interventions in adult patients with cancer found that they had significant effects on existential wellbeing (*g* = 0.52; CI[0.13; 0.91; *k* = 10; I^2^ = 85%) [49]. Routine screening to assess spiritual wellbeing and spiritual needs is recommended in the oncology setting to facilitate appropriate referral of patients to spiritual care services where desired.

### 5.1 Study Limitations

A minority of patients completed information at both time points, which reduced the power of our findings, and older patients, less educated patients, and those with English as a second language, were underrepresented in our data. Nonetheless, this study gives helpful information about the psychological impact of genomic test results and the role of spiritual wellbeing in advanced cancer patients. The generalizability to other religio‐cultural settings of the findings is not known.

### 5.2 Clinical Implications

This study found that CGP results do not exacerbate FCP. It also found that high levels of spiritual wellbeing are associated with reduced fear of cancer progression. As spiritual wellbeing can be improved with spiritual care, it should be routinely offered to cancer patients.

## 6 Conclusions

Assessment of advanced cancer patients undergoing CGP seeking novel therapeutic options found that the levels of FCP and spiritual wellbeing were not impacted by test results but influenced a number of patient outcomes. Further work to examine the impact of spiritual care on patient spiritual wellbeing in such cohorts is recommended as a possible means to reduce fear of cancer progression.

## Funding

The PiGeOn Project was funded by a National Health and Medical Research Council (NHMRC) of Australia Project Grant (ID 1124749). Investigators received the following support during the project: PB, NHMRC Senior Principal Research Fellowship; MCB, Post‐Doctoral Research Fellowship from the Cancer Institute NSW; MLB, Cancer Institute NSW Career Development Fellowship; DMT, NHMRC Principal Research Fellowship. No funding body had any input in the design of the study, or collection, analysis, and interpretation of data or in writing the manuscript.

## Conflicts of Interest

The authors declare no conflicts of interest.

## Supporting information


Supporting Information S1

**Table S1:** Demographic characteristics by completeness (Total *N* = 1186).

**Table S2:** Pearson correlation coefficients between FACIT‐Sp12 subscales at baseline and time 1 (*n* = 397).

## Data Availability

The data that support the findings of this study are available on request from the corresponding author. The data are not publicly available due to privacy or ethical restrictions.
