# Supplementary material for: Impact of Spiritual Wellbeing in Advanced Cancer Patients Receiving Genomic Test Results
Source: Psychooncology. 2026 Apr 23;35:e70471. doi: 10.1002/pon.70471 (PMC13106103; doi:10.1002/pon.70471)

**SUPPLEMENTARY ONLINE FILES**

**Figure 1: Frequencies of each response at baseline and time 1 by subscale.**

1. **Item frequencies within the meaning subscale**

**
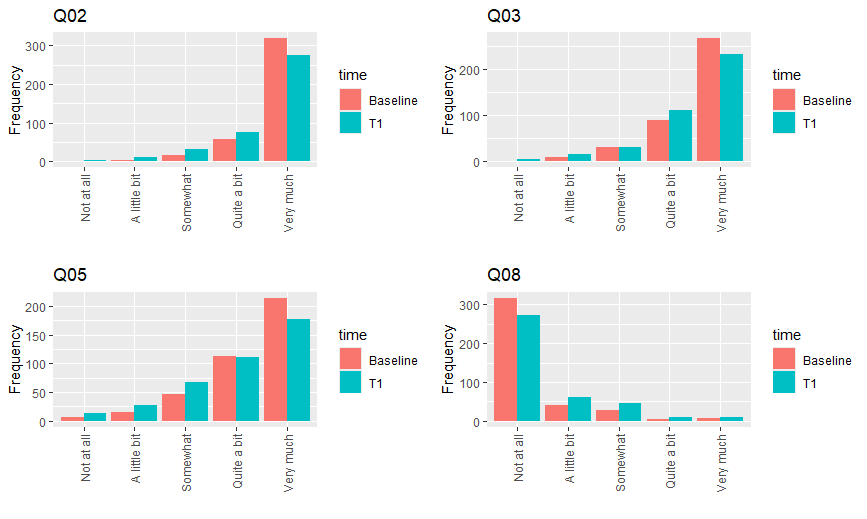
**

1. **Items frequencies in the peace subscale**


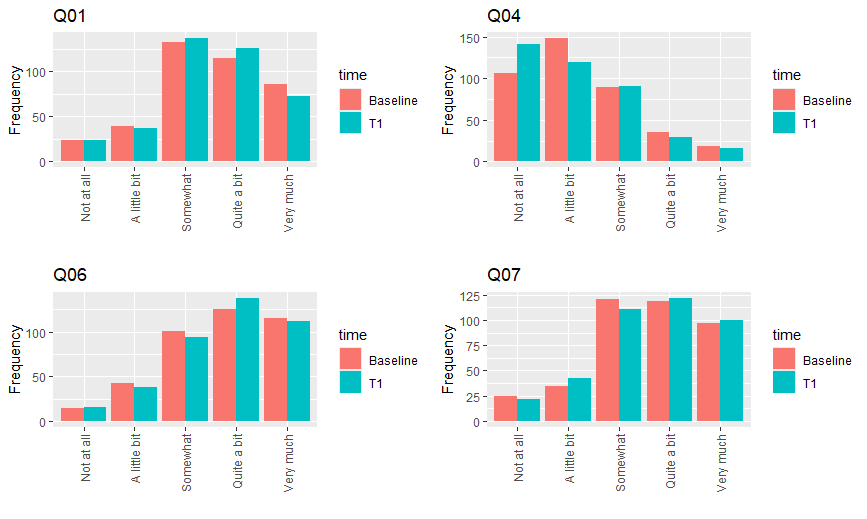


1. **Items frequencies in the faith subscale**


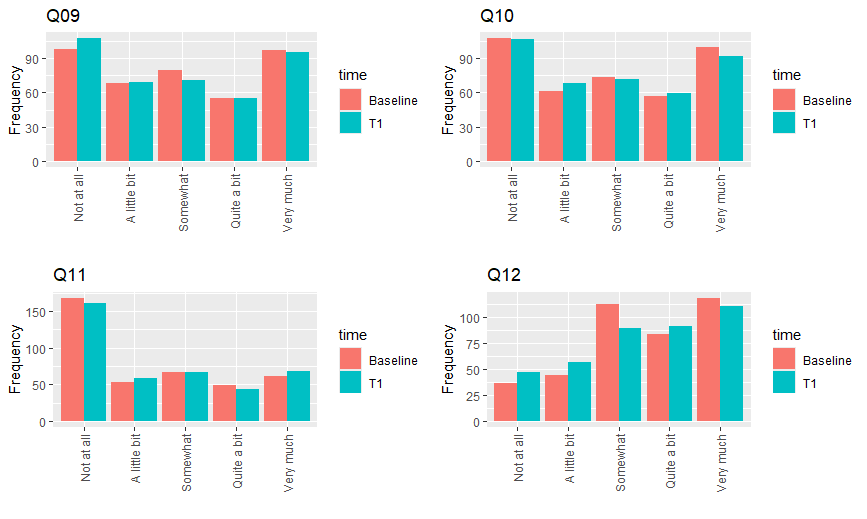


**Figure 2:** **Graphs of reliability for each subscale at baseline.** The y-axis represents reliability of the subscale. The x-axis represents a latent score of the subscale, where 0 indicates an individual with an average level of the subscale, -1 indicates -1 standard deviation (SD) below the average level of the subscale, and 1 indicates 1 SD above the average level.


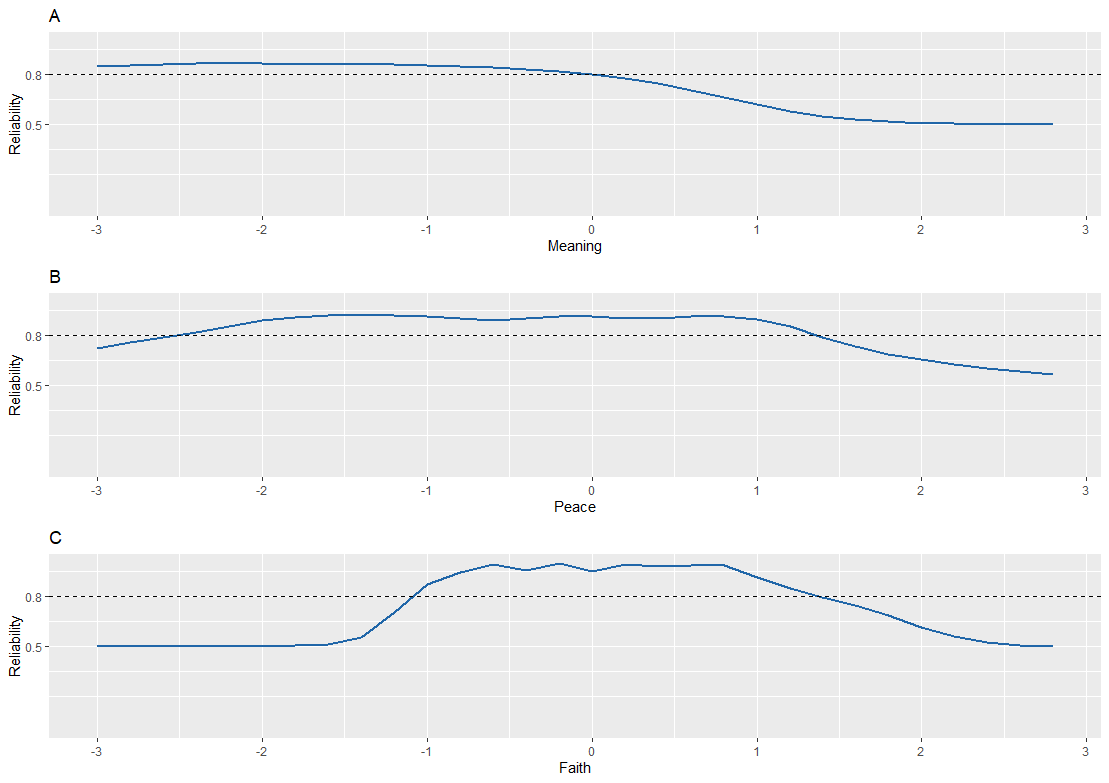

Supplement: Supplementary file 1 — Supporting Information S1 [file PON-35-e70471-s002.docx]
