# Supplementary material for: Impact of Spiritual Wellbeing in Advanced Cancer Patients Receiving Genomic Test Results
Source: Psychooncology. 2026 Apr 23;35:e70471. doi: 10.1002/pon.70471 (PMC13106103; doi:10.1002/pon.70471)
Supplement: Supplementary file 2 — Table S1: Demographic characteristics by completeness (Total N = 1186). [file PON-35-e70471-s003.docx]

**Supplementary Table 1: Demographic characteristics by completeness (Total N = 1186)**

| **Characteristics** | **Excluded from study (n = 789)** | **Included (n = 397)** | **p-value** |
| --- | --- | --- | --- |
| **Sex** (n,%) |  |  |  |
| Male | 390 (68.9) | 176 (31.1) | 0.1092 |
| Female | 399 (64.4) | 221 (35.7) |  |
| **Age** (m,sd) | 56.98 (13.75) | 54.98 (14.19) | 0.0206 |
| **Education Level** (n,%) |  |  |  |
| Primary School | 10 (71.4) | 4 (28.6) | 0.0021 |
| Year 7 or 8 | 13 (43.3) | 17 (56.7) |  |
| Year 9 or 10 | 118 (61.50) | 74 (38.5) |  |
| Year 11 or 12 | 150 (71.8) | 59 (28.2) |  |
| University | 325 (65.4) | 172 (34.6) |  |
| Vocational training | 158 (69) | 71 (31) |  |
| **Cultural and linguistically diverse background** (n,%) |  |  |  |
| Yes | 194 (75.5) | 63 (24.5) | 0.0007 |
| No | 593 (64) | 334 (36) |  |
| Missing (n = 2) |  |  |  |
| **Socioeconomic status (SEIFA)** |  |  |  |
| Level 1 | 87 (69.6) | 38 (30.4) | 0.3279 |
| Level 2 | 126 (65.3) | 67 (34.7) |  |
| Level 3 | 148 (67.3) | 72 (32.7) |  |
| Level 4 | 145 (71.4) | 58 (28.6) |  |
| Level 5 | 283 (63.6) | 162 (36.4) |  |
| **Accessibility/Remoteness Index of Australia (ARIA)** |  |  |  |
| Major City | 590 (68.1) | 277 (32) | 0.3325 |
| Inner regional | 124 (62) | 76 (38) |  |
| Outer regional | 66 (62.9) | 39 (37.1) |  |
| Remote | 9 (64.3) | 5 (35.7) |  |
| **Previous family cancer clinic visit** |  |  |  |
| Yes | 72 (61.5) | 45 (38.5) | 0.066 |
| No | 694 (67.3) | 337 (32.7) |  |
| Missing (n = 6) |  |  |  |
| **Children** |  |  |  |
| Yes | 602 (65.6) | 316 (34.4) | 0.308 |
| No | 185 (70.1) | 79 (29.9) |  |
| Missing (n = 4) |  |  |  |
