# Supplementary material for: Impact of Spiritual Wellbeing in Advanced Cancer Patients Receiving Genomic Test Results
Source: Psychooncology. 2026 Apr 23;35:e70471. doi: 10.1002/pon.70471 (PMC13106103; doi:10.1002/pon.70471)
Supplement: Supplementary file 3 — Table S2: Pearson correlation coefficients between FACIT‐Sp12 subscales at baseline and time 1 (n = 397). [file PON-35-e70471-s001.docx]

**Supplementary Table 2: Pearson correlation coefficients between FACIT-Sp12 subscales at baseline and time 1 (n = 397)**

|  | Meaning (baseline) | Peace (baseline) | Faith (baseline) | Meaning (time 1) | Peace (time 1) | Faith (time 1) |
| --- | --- | --- | --- | --- | --- | --- |
| Meaning (baseline) |  |  |  |  |  |  |
| Peace (baseline) | 0.36 |  |  |  |  |  |
| Faith (baseline) | 0.14 | 0.38 |  |  |  |  |
| Meaning (time 1) | 0.56 | 0.34 | 0.17 |  |  |  |
| Peace (time 1) | 0.3 | 0.64 | 0.33 | 0.61 |  |  |
| Faith (time 1) | 0.15 | 0.34 | 0.79 | 0.32 | 0.49 |  |
